# Chloridotetra­pyridine­copper(II) dicyanamidate pyridine disolvate

**DOI:** 10.1107/S1600536811016187

**Published:** 2011-05-07

**Authors:** Susanne Wöhlert, Mario Wriedt, Inke Jess, Christian Näther

**Affiliations:** a Institut für Anorganische Chemie, Christian-Albrechts-Universität Kiel, Max-Eyth-Strasse 2, 24118 Kiel, Germany; b Departement of Chemistry, Texas A&M University, College Station, Texas 77843, USA

## Abstract

In the crystal structure of the title compound, [CuCl(C_5_H_5_N)_4_][N(CN)_2_]·2C_6_H_5_N, the copper(II) cations are coordinated by one chloride anion and four *N*-bonded pyridine ligands into discrete complexes. The copper(II) cation shows a square-pyramidal coordination environment, with the chloride anion in the apical position. However, there is one additional chloride anion at 3.0065 (9) Å, leading to a disorted octa­hedral coordination mode for copper. The copper(II) cation, the chloride ligand and the central N atom of the dicyanamide anion are located on twofold rotation axes. Two pyridine solvent molecules are observed in general positions.

## Related literature

For background to this work, see: Wriedt *et al.* (2009*a* ▶,*b* ▶). For structures of transition metal dicyanamides, see: Wriedt & Näther (2011 ▶) and for a related structure, see: Potočňák *et al.* (2006) ▶. For a description of the Cambridge Structural Database, see: Allen (2002 ▶).
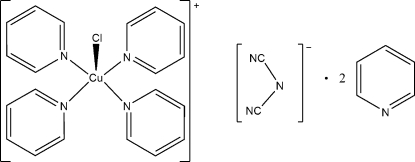


## Experimental

### 

#### Crystal data

[CuCl(C_5_H_5_N)_4_](C_2_N_3_)·2C_6_H_5_N
*M* *_r_* = 639.64
Orthorhombic, 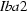

*a* = 15.2859 (6) Å
*b* = 17.6577 (9) Å
*c* = 11.4818 (9) Å
*V* = 3099.1 (3) Å^3^
*Z* = 4
Mo *K*α radiation
μ = 0.83 mm^−1^
*T* = 170 K
0.48 × 0.18 × 0.08 mm

#### Data collection

Stoe IPDS-1 diffractometer
Absorption correction: numerical (*X-SHAPE*; Stoe & Cie, 1998) ▶ *T*_min_ = 0.825, *T*_max_ = 0.941
16623 measured reflections
3708 independent reflections
3220 reflections with *I* > 2σ(*I*)
*R*_int_ = 0.046

#### Refinement

*R*[*F* ^2^ > 2σ(*F* ^2^)] = 0.034
*wR*(*F* ^2^) = 0.093
*S* = 1.03
3708 reflections
198 parameters
1 restraint
H-atom parameters constrained
Δρ_max_ = 0.71 e Å^−3^
Δρ_min_ = −0.56 e Å^−3^
Absolute structure: Flack (1983 ▶), 1771 Friedel pairs
Flack parameter: 0.00 (2)

### 

Data collection: *IPDS* (Stoe & Cie, 1998) ▶; cell refinement: *IPDS* ▶; data reduction: *IPDS* ▶; program(s) used to solve structure: *SHELXS97* (Sheldrick, 2008 ▶); program(s) used to refine structure: *SHELXL97* (Sheldrick, 2008 ▶); molecular graphics: *XP* in *SHELXTL* (Sheldrick, 2008 ▶); software used to prepare material for publication: *CIFTAB* in *SHELXTL*.

## Supplementary Material

Crystal structure: contains datablocks I, global. DOI: 10.1107/S1600536811016187/im2272sup1.cif

Structure factors: contains datablocks I. DOI: 10.1107/S1600536811016187/im2272Isup2.hkl

Additional supplementary materials:  crystallographic information; 3D view; checkCIF report

## Figures and Tables

**Fig. 1.**
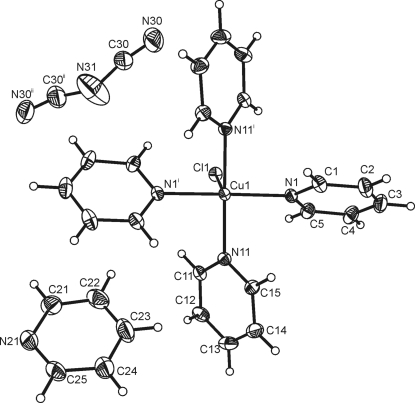
: Crystal structure of the title compound with labelling and displacement ellipsoids drawn at the 50% probability level. Symmetry codes: i = -x+1, -y+1, z; ii = -x+2, -y+1, z.

**Fig. 2.**
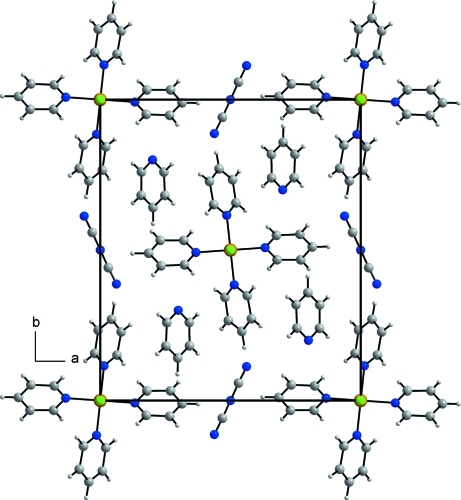
: Crystal structure of the title compound with view along the crystallographic c-axis.

## supplementary crystallographic information

### Comment

In our recent work we have shown that thermal decomposition reactions are an elegant route for the discovery and preparation of new ligand-deficient coordination polymers based on transition metal thiocyanates and N-donor ligands (Wriedt *et al.* 2009*a*,*b*). In further investigations we have shown that new transition metal dicyanamides can also be prepared by this route (Wriedt & Näther, 2011). In order to prepare new precursors with pyridine ligands we have reacted copper (II) chloride, sodium dicyanamide and pyridine. In this reaction single crystals of the title compound were obtained by accident, which were characterized by single crystal X-ray diffraction.

In the crystal structure of the title compound each copper (II) cation is coordinated by one chloride anion and by four pyridine ligands into discrete complexes which are located on a 2-fold rotation axis (Fig. 1). The copper(II) cations are in a slightly distorted square pyramidal coordination with two Cu—N distances of 2.0511 (16) Å, two Cu—N distances of 2.0374 (16) Å and one Cu—Cl distance of 2.7344 (9) Å. The angles around the copper(II) cations ranges from 87.76 (6) ° to 91.59 (6) ° (Tab. 1). There is one additional chloride anion at 3.0065 (9) Å. If this distance is considered in copper coordination the coordination polyhedron can be described as a slightly disorted octahedron. The discrete complexes are stacked into columns that elongate in the direction of the *c*-axis (Fig. 2). Between these columns additional pyridine molecules as well as non-coordinated dicyanamide anions are located (Fig. 2). The distances between the discrete complexe cations [CuCl(pyridine)]^+^ and the non-coordinated [N(CN_2_)]^-^ anions amounts to 7.469 (3) Å and the shortest Cu···Cu distances amount to 5.7409 (5) Å.

It must be noted that according to a search in the CCDC database (ConQuest Ver.1.12.2010) (Allen, 2002) compounds with copper (II) cations, chloro anions and dicyanamide are unkown but with 1,10-phenanthroline one compound is reported (Potočňák *et al.*, 2006).

### Experimental

Copper (II) chloride dihydrate (CuCl_2_ × 2 H_2_O) and sodium dicyanamide (Na(dca)) were obtained from Alfa Aesar and pyridine was obtained from Riedel de Haen. All chemicals were used without further purification. 0.25 mmol (42.62 mg) CuCl_2_ × 2 H_2_O and 0.5 mmol (44.51 mg) Na(dca) were reacted in 0.5 ml pyridine. Blue single crystals of the title compound were obtained after one day.

### Refinement

H atoms were positioned with idealized geometry and were refined isotropically with *U*_iso_(H) = 1.2 *U*_eq_(C) and C—H distances of 0.95 Å using a riding model. The absolute structure was determined on the basis of 1740 Friedel pairs but the crystal investigated was racemically twinned. Therefore, a twin refinement was performed (BASF parameter: 0.25 (2).

### Crystal data

**Table:**

[CuCl(C_5_H_5_N)_4_](C_2_N_3_)·2C_6_H_5_N	*F*(000) = 1324
*M**_r_* = 639.64	*D*_x_ = 1.371 Mg m^−^^3^
Orthorhombic, *I**b**a*2	Mo *K*α radiation, λ = 0.71073 Å
Hall symbol: I 2 -2c	Cell parameters from 16623 reflections
*a* = 15.2859 (6) Å	θ = 2.7–28°
*b* = 17.6577 (9) Å	µ = 0.83 mm^−^^1^
*c* = 11.4818 (9) Å	*T* = 170 K
*V* = 3099.1 (3) Å^3^	Block, blue
*Z* = 4	0.48 × 0.18 × 0.08 mm

### Data collection

**Table:**

Stoe IPDS-1 diffractometer	3708 independent reflections
Radiation source: fine-focus sealed tube	3220 reflections with *I* > 2σ(*I*)
graphite	*R*_int_ = 0.046
φ scans	θ_max_ = 28.0°, θ_min_ = 2.7°
Absorption correction: numerical (*X-SHAPE*; Stoe & Cie, 1998)	*h* = −20→20
*T*_min_ = 0.825, *T*_max_ = 0.941	*k* = −23→23
16623 measured reflections	*l* = −15→15

### Refinement

**Table:**

Refinement on *F*^2^	Hydrogen site location: inferred from neighbouring sites
Least-squares matrix: full	H-atom parameters constrained
*R*[*F*^2^ > 2σ(*F*^2^)] = 0.034	*w* = 1/[σ^2^(*F*_o_^2^) + (0.0648*P*)^2^] where *P* = (*F*_o_^2^ + 2*F*_c_^2^)/3
*wR*(*F*^2^) = 0.093	(Δ/σ)_max_ = 0.001
*S* = 1.03	Δρ_max_ = 0.71 e Å^−^^3^
3708 reflections	Δρ_min_ = −0.56 e Å^−^^3^
198 parameters	Extinction correction: *SHELXL97* (Sheldrick, 2008), Fc^*^=kFc[1+0.001xFc^2^λ^3^/sin(2θ)]^-1/4^
1 restraint	Extinction coefficient: 0.0056 (6)
Primary atom site location: structure-invariant direct methods	Absolute structure: Flack (1983), 1740 Friedel pairs
Secondary atom site location: difference Fourier map	Flack parameter: 0.00 (2)

### Special details

**Table:**

Geometry. All esds (except the esd in the dihedral angle between two l.s. planes) are estimated using the full covariance matrix. The cell esds are taken into account individually in the estimation of esds in distances, angles and torsion angles; correlations between esds in cell parameters are only used when they are defined by crystal symmetry. An approximate (isotropic) treatment of cell esds is used for estimating esds involving l.s. planes.
Refinement. Refinement of F^2^ against ALL reflections. The weighted R-factor wR and goodness of fit S are based on F^2^, conventional R-factors R are based on F, with F set to zero for negative F^2^. The threshold expression of F^2^ > 2sigma(F^2^) is used only for calculating R-factors(gt) etc. and is not relevant to the choice of reflections for refinement. R-factors based on F^2^ are statistically about twice as large as those based on F, and R- factors based on ALL data will be even larger.

### Fractional atomic coordinates and isotropic or equivalent isotropic displacement parameters (Å^2^)

**Table:**

	*x*	*y*	*z*	*U*_iso_*/*U*_eq_	
Cu1	0.5000	0.5000	0.78178 (3)	0.01901 (12)	
Cl1	0.5000	0.5000	1.01993 (7)	0.01916 (17)	
N1	0.51436 (10)	0.38455 (9)	0.77683 (18)	0.0175 (3)	
C2	0.49213 (17)	0.26597 (16)	0.6798 (2)	0.0291 (6)	
H2	0.4664	0.2383	0.6177	0.035*	
C3	0.54374 (17)	0.22991 (13)	0.7620 (2)	0.0310 (5)	
H3	0.5538	0.1769	0.7570	0.037*	
C4	0.58055 (16)	0.27172 (12)	0.8518 (2)	0.0288 (5)	
H4	0.6160	0.2479	0.9091	0.035*	
C5	0.56461 (14)	0.34873 (12)	0.8560 (2)	0.0222 (4)	
H5	0.5900	0.3775	0.9172	0.027*	
N11	0.63287 (11)	0.50796 (9)	0.77505 (18)	0.0169 (3)	
C11	0.67644 (14)	0.54903 (11)	0.8549 (2)	0.0193 (4)	
H11	0.6440	0.5768	0.9112	0.023*	
C12	0.76675 (16)	0.55225 (14)	0.8580 (2)	0.0257 (5)	
H12	0.7960	0.5824	0.9144	0.031*	
C13	0.81395 (14)	0.51051 (14)	0.7770 (3)	0.0294 (5)	
H13	0.8761	0.5114	0.7777	0.035*	
C14	0.76982 (15)	0.46773 (14)	0.6956 (2)	0.0264 (5)	
H14	0.8010	0.4386	0.6397	0.032*	
C15	0.67906 (14)	0.46793 (13)	0.6968 (2)	0.0210 (4)	
H15	0.6485	0.4388	0.6404	0.025*	
C1	0.47896 (16)	0.34335 (13)	0.6904 (2)	0.0225 (4)	
H1	0.4435	0.3683	0.6342	0.027*	
N21	0.79940 (15)	0.79986 (11)	0.5241 (2)	0.0355 (5)	
C21	0.7555 (2)	0.75937 (16)	0.6032 (2)	0.0369 (6)	
H21	0.7249	0.7859	0.6625	0.044*	
C22	0.7521 (2)	0.68134 (18)	0.6039 (3)	0.0413 (7)	
H22	0.7193	0.6552	0.6615	0.050*	
C23	0.79745 (19)	0.64167 (15)	0.5190 (3)	0.0444 (7)	
H23	0.7970	0.5879	0.5178	0.053*	
C24	0.8428 (2)	0.68182 (16)	0.4368 (3)	0.0401 (7)	
H24	0.8741	0.6566	0.3767	0.048*	
C25	0.8419 (2)	0.75980 (16)	0.4435 (3)	0.0378 (6)	
H25	0.8740	0.7870	0.3864	0.045*	
N30	0.93807 (17)	0.38709 (14)	0.5329 (4)	0.0628 (8)	
C30	0.96901 (19)	0.44145 (16)	0.5161 (4)	0.0527 (9)	
N31	1.0000	0.5000	0.4511 (5)	0.0835 (19)	

### Atomic displacement parameters (Å^2^)

**Table:**

	*U*^11^	*U*^22^	*U*^33^	*U*^12^	*U*^13^	*U*^23^
Cu1	0.01452 (16)	0.01243 (16)	0.0301 (2)	−0.00084 (13)	0.000	0.000
Cl1	0.0229 (3)	0.0194 (3)	0.0151 (4)	0.0001 (3)	0.000	0.000
N1	0.0189 (8)	0.0146 (7)	0.0188 (7)	−0.0016 (6)	0.0009 (7)	0.0003 (7)
C2	0.0405 (16)	0.0208 (12)	0.0259 (13)	−0.0078 (10)	0.0045 (9)	−0.0045 (7)
C3	0.0379 (13)	0.0177 (10)	0.0375 (15)	0.0035 (10)	0.0098 (10)	0.0000 (8)
C4	0.0334 (12)	0.0230 (11)	0.0299 (11)	0.0106 (10)	0.0009 (10)	0.0057 (9)
C5	0.0251 (11)	0.0206 (9)	0.0210 (9)	0.0034 (9)	−0.0008 (8)	0.0023 (8)
N11	0.0163 (6)	0.0175 (8)	0.0169 (8)	−0.0007 (6)	−0.0013 (7)	−0.0001 (6)
C11	0.0228 (10)	0.0172 (9)	0.0179 (9)	−0.0039 (8)	0.0000 (8)	−0.0005 (8)
C12	0.0233 (11)	0.0296 (11)	0.0241 (10)	−0.0074 (9)	−0.0035 (9)	0.0011 (9)
C13	0.0178 (8)	0.0386 (13)	0.0317 (11)	−0.0009 (9)	0.0028 (11)	0.0067 (10)
C14	0.0220 (12)	0.0289 (12)	0.0282 (11)	0.0027 (10)	0.0037 (9)	0.0021 (10)
C15	0.0214 (11)	0.0214 (10)	0.0201 (9)	0.0007 (9)	0.0021 (9)	−0.0017 (8)
C1	0.0274 (11)	0.0200 (10)	0.0202 (8)	−0.0050 (9)	−0.0013 (9)	−0.0002 (9)
N21	0.0417 (12)	0.0274 (9)	0.0375 (10)	0.0003 (9)	0.0084 (11)	0.0080 (9)
C21	0.0384 (15)	0.0408 (16)	0.0314 (12)	0.0044 (13)	0.0074 (9)	0.0092 (10)
C22	0.0395 (15)	0.0433 (16)	0.0412 (15)	−0.0056 (13)	0.0035 (11)	0.0181 (12)
C23	0.0511 (16)	0.0258 (11)	0.0562 (17)	−0.0071 (12)	−0.0008 (14)	0.0103 (12)
C24	0.0467 (19)	0.0304 (13)	0.0431 (14)	−0.0018 (13)	0.0081 (11)	0.0023 (11)
C25	0.0447 (17)	0.0327 (13)	0.0362 (12)	−0.0063 (12)	0.0129 (11)	0.0090 (11)
N30	0.0391 (13)	0.0320 (12)	0.117 (2)	0.0062 (11)	−0.0058 (16)	0.0096 (19)
C30	0.0252 (11)	0.0321 (15)	0.101 (3)	−0.0010 (12)	0.0075 (16)	−0.0040 (16)
N31	0.105 (5)	0.091 (4)	0.054 (3)	−0.054 (3)	0.000	0.000

### Geometric parameters (Å, °)

**Table:**

Cu1—N11	2.0374 (16)	C13—C14	1.378 (4)
Cu1—N11^i^	2.0374 (16)	C13—H13	0.9500
Cu1—N1	2.0511 (16)	C14—C15	1.387 (3)
Cu1—N1^i^	2.0511 (16)	C14—H14	0.9500
Cu1—Cl1	2.7344 (9)	C15—H15	0.9500
N1—C1	1.345 (3)	C1—H1	0.9500
N1—C5	1.348 (3)	N21—C25	1.333 (4)
C2—C3	1.385 (4)	N21—C21	1.337 (3)
C2—C1	1.386 (4)	C21—C22	1.379 (4)
C2—H2	0.9500	C21—H21	0.9500
C3—C4	1.387 (3)	C22—C23	1.386 (5)
C3—H3	0.9500	C22—H22	0.9500
C4—C5	1.382 (3)	C23—C24	1.368 (4)
C4—H4	0.9500	C23—H23	0.9500
C5—H5	0.9500	C24—C25	1.379 (4)
N11—C15	1.344 (3)	C24—H24	0.9500
N11—C11	1.346 (3)	C25—H25	0.9500
C11—C12	1.382 (3)	N30—C30	1.087 (4)
C11—H11	0.9500	C30—N31	1.360 (4)
C12—C13	1.389 (4)	N31—C30^ii^	1.360 (4)
C12—H12	0.9500		
			
N11—Cu1—N11^i^	175.66 (12)	C11—C12—H12	120.7
N11—Cu1—N1	87.76 (6)	C13—C12—H12	120.7
N11^i^—Cu1—N1	92.12 (6)	C14—C13—C12	119.39 (19)
N11—Cu1—N1^i^	92.12 (6)	C14—C13—H13	120.3
N11^i^—Cu1—N1^i^	87.76 (6)	C12—C13—H13	120.3
N1—Cu1—N1^i^	176.83 (12)	C13—C14—C15	118.8 (2)
N11—Cu1—Cl1	92.17 (6)	C13—C14—H14	120.6
N11^i^—Cu1—Cl1	92.17 (6)	C15—C14—H14	120.6
N1—Cu1—Cl1	91.59 (6)	N11—C15—C14	122.2 (2)
N1^i^—Cu1—Cl1	91.59 (6)	N11—C15—H15	118.9
C1—N1—C5	118.27 (18)	C14—C15—H15	118.9
C1—N1—Cu1	121.01 (15)	N1—C1—C2	122.6 (2)
C5—N1—Cu1	120.58 (15)	N1—C1—H1	118.7
C3—C2—C1	118.4 (2)	C2—C1—H1	118.7
C3—C2—H2	120.8	C25—N21—C21	115.6 (2)
C1—C2—H2	120.8	N21—C21—C22	123.9 (3)
C2—C3—C4	119.5 (2)	N21—C21—H21	118.1
C2—C3—H3	120.2	C22—C21—H21	118.1
C4—C3—H3	120.2	C21—C22—C23	118.8 (2)
C5—C4—C3	118.6 (2)	C21—C22—H22	120.6
C5—C4—H4	120.7	C23—C22—H22	120.6
C3—C4—H4	120.7	C24—C23—C22	118.4 (2)
N1—C5—C4	122.5 (2)	C24—C23—H23	120.8
N1—C5—H5	118.7	C22—C23—H23	120.8
C4—C5—H5	118.7	C23—C24—C25	118.3 (3)
C15—N11—C11	118.64 (17)	C23—C24—H24	120.9
C15—N11—Cu1	120.85 (14)	C25—C24—H24	120.9
C11—N11—Cu1	120.31 (15)	N21—C25—C24	125.0 (2)
N11—C11—C12	122.3 (2)	N21—C25—H25	117.5
N11—C11—H11	118.9	C24—C25—H25	117.5
C12—C11—H11	118.9	N30—C30—N31	156.9 (5)
C11—C12—C13	118.7 (2)	C30^ii^—N31—C30	113.5 (5)

Symmetry codes: (i) −*x*+1, −*y*+1, *z*; (ii) −*x*+2, −*y*+1, *z*.
